# Melatonin attenuates ovarian ischemia reperfusion injury in rats by decreasing oxidative stress index and peroxynitrite

**DOI:** 10.3906/sag-2004-135

**Published:** 2020-10-22

**Authors:** Şenol KALYONCU, Bülent YILMAZ, Mustafa DEMİR, Meltem TUNCER, Zehra BOZDAĞ, Onur İNCE, Mehmet Akif BOZDAYI, Hasan ULUSAL, Seyithan TAYSİ

**Affiliations:** 1 Department of Obstetrics and Gynecology, TOBB ETU University Hospital, Ankara Turkey; 2 Department of Obstetrics and Gynecology, Faculty of Medicine, Recep Tayyip Erdoğan University, Rize Turkey; 3 Department of Obstetrics and Gynecology, ANKA Hospital, Gaziantep Turkey; 4 Department of Physiology, Faculty of Medicine, Hacettepe University, Ankara Turkey; 5 Department of Pathology, Faculty of Medicine, Gaziantep University, Gaziantep Turkey; 6 Department of Obstetrics and Gynecology, Faculty of Medicine, Health Science University, Kütahya Turkey; 7 Department of Biochemistry, Faculty of Medicine, Gaziantep University, Gaziantep Turkey

**Keywords:** Ischemia reperfusion injury, rat ovarian torsion, melatonin, oxidative stress index, peroxynitrite

## Abstract

**Background/aim:**

To evaluate the protective effect of melatonin on ovarian ischemia reperfusion injury in a rat model.

**Materials and methods:**

Forty-eight rats were separated equally into 6 groups. Group 1: sham; Group 2: surgical control with 3-h bilateral ovarian torsion and detorsion; Group 3: intraperitoneal 5% ethanol (1 mL) just after detorsion (as melatonin was dissolved in ethanol); Group 4: 10 mg/kg intraperitoneal melatonin 30 min before 3-h torsion; Group 5:10 mg/kg intraperitoneal melatonin just after detorsion; Group 6:10 mg/kg intraperitoneal melatonin 30 min before torsion and just after detorsion. Both ovaries and blood samples were obtained 7 days after detorsion for histopathological and biochemical analysis.

**Results:**

In Group 1, serum levels of total oxidant status (TOS) (μmol H2O2 equivalent/g wet tissue)were significantly lower than in Group2 (P = 0.0023), while tissue TOS levels were lower than in Group 3 (P = 0.0030). Similarly, serum and tissue levels of peroxynitrite in Group 6were significantly lower than those ofGroup 2 (P = 0.0023 and P = 0.040, respectively). Moreover, serum oxidative stress index (OSI) (arbitrary unit) levels were significantly increased in Group 2 when compared to groups 1 and 6 (P = 0.0023 and P= 0.0016, respectively) and in Group 3 with respect to groups 1, 4, 5, and 6 (P = 0.0023, P = 0.0026, P = 0.0008, and P = 0.0011, respectively). Furthermore, there was a significant decrease in histopathological scores including follicular degeneration, vascular congestion, hemorrhage, and inflammation in the melatonin and sham groups in comparison with control groups. Additionally, primordial follicle count was significantly higher in Group 6 than in Group 2 (P = 0.0002).

**Conclusion:**

Melatonin attenuates ischemia reperfusion damage in a rat torsion/detorsion model by improving histopathological and biochemical findings including OSI and peroxynitrite.

## 1. Introduction

Twisting of the ovary along with the fallopian tube is referred to as adnexal torsion; it may affect females of all ages, particularly women of reproductive age from 20 to 40 years of age [1]. The most common symptom of ovarian torsion is acute onset of lower abdominal pain, followed by nausea and vomiting. It presents clinically as a gynecologic surgical emergency and often compromises ovarian lymphatic and blood supply. Therefore, urgent diagnosis and treatment is crucial to preserve ovarian function and to prevent other related morbidities in patients with ovarian torsion. 

The most common method of treatment is laparoscopy, and the main objective of the intraoperative evaluation is to confirm the diagnosis of torsion and evaluate the viability of the ovarian tissue. Most twisted ovaries are considered potentially viable and can be preserved by detorsion unless there is an ovarian mass with suspicionof malignancy or the patient is postmenopausal, in which casesalpingo-oophorectomy is performed [2]. The blood supply to the twisted ovary is sufficient for survival of follicles despite its necrotic black bluish appearance [3].

Untwisting of the twisted ovary reestablishes the blood supply; however, the return of blood flow can also result in further ovarian damage and complications, termed ischemia reperfusion injury [4]. Recently, many pharmacological agents have been found to alleviate the effects of ischemia reperfusion injury in animal models, due to their antioxidant and antiinflammatory effects [5–8]. 

Melatonin (5 methoxy-N-acetyltryptamine), discovered and isolated from bovine pineal gland by Aaron Lerner in 1958,is produced in many organs, mainly by the pineal gland [9,10]. The circadian production of melatonin explains its regulatory action on circadian rhythms such as body temperature or sleep-wake cycles and neuroendocrine rhythms [11,12]. Moreover, it has antioxidant and antiinflammatory properties and critical effects on many diseases, including asthma [13], diabetic retinopathy [14), depression [15], infection [16], and endometriosis [17]. However, there have been only 3 studies in the literature showing preventive effects of melatonin on ischemia reperfusion injury of the rat ovarian torsion/detorsion model [18–20]. In addition, it has also been investigated in cat ovarian [21] and rat uterine [22] torsion/detorsion models.

The objective of this study was to investigate the effectiveness of melatonin in preventing ovarian ischemia reperfusion injury in a rat model through biochemical and histopathological analysis.

## 2. Materials and methods

### 2.1. Animal care 

A total of 48 female adult Wistar albino rats weighing 150–250 g and aged 10–15 weeks were included in this study, which was performed at the Hacettepe University Department of Physiology Animal Research Laboratory. All experimental procedures were carried out once the study protocol was approved by the Hacettepe University Ethical Committee on Animal Research (Approval No. 2019/08-02), with a commentary advising that both the sham and surgical control groups (16 rats in total) should be the used in another similar study of ours (unpublished) performed at the same time period to decrease the number of rats used. The animals were fed ad libitum and kept in a temperature and humidity-controlled environment (22 ± 2 °C) with a cycle of 12 h of darkness followed by 12 h of light.

### 2.2. Surgical procedures and groups

Animals were allocated randomly into 6 groups. Group 1 (n = 8, sham group): the abdominal wall was kept open for 3 h and then closed. Ovarian torsion was not performed. Rats were followed-up for 1 week after the first operation without any medication. Group 2 (n = 8, torsion/detorsion + control group): bilateral ovarian torsion was performed; 3 h after ischemia, detorsion was applied. No treatment agent was given to the rats during the follow-up period of 1 week. Group 3 (n = 8, torsion/detorsion + ethanol group): bilateral ovarian torsion was performed; 3 h after ischemia, detorsion was applied. A single dose of 1-mL 5% ethanol solution was given intraperitoneally (i.p.) to all rats just after detorsion and administered daily for 1week (as melatonin was dissolved in ethanol solution, it was aimed to see the effects of ethanol itself on ischemia reperfusion injury in Group 3).Group 4(n = 8, torsion/detorsion + melatonin prophylaxis only group): 30 min before the induction of ovarian torsion, rats were given a single dose of 10 mg/kg i.p.melatonin (melatonin powder, Alfa Aesar GmbH & Co KG, Karlsruhe, Germany). No maintenance treatment was applied after detorsion or during the follow-up period of 1 week. Melatonin in dry powder form was dissolved in 99% ethanol and then diluted in saline just before injection with final ethanol and melatonin concentrations of 5%and 10 mg, respectively, per mL,as in our previous studies [17]. Group 5 (n = 8, torsion/detorsion + melatonin prophylaxis plus maintenance group): a dosage of 10 mg/kg i.p. melatonin was given to each rat 30 min before the induction of ovarian torsion, and repeated just after detorsion preceding 3 h of ischemia. A 10 mg/kg i.p. dose of melatonin was administered daily for 1 week. Group 6 (n = 8, torsion/detorsion + melatonin maintenanceonly group): a single dose of 10 mg/kg i.p. melatonin was administered to each rat just after the ovarian detorsion preceding 3 h of ischemia, and repeated daily during the 1-week period, postoperatively. 

Rats were anesthetized withan i.p. injection of a combination of ketamine hydrochloride (50 mg/kg Ketalar; Eczacıbaşı Holding, İstanbul, Turkey) and xylazine hydrochloride (10 mg/kg Rompun; Bayer Türk Kimya Sanayii Ltd. Şti., İstanbul, Turkey).Once the abdomen was shaved and prepared with a povidone-iodine scrub, a laparotomy was performed by making a 2-cm longitudinal incision in the midline area of the lower abdomen, and the uterine horns and adnexa were viewed. Ischemia was induced for 3 h using a torsion model conducted by applying and rotating atraumatic vascular clips 360° clockwise to the vascular pedicle 1 cm above and below each ovary bilaterally. The abdominal wall including muscle aponeurotic plane and skin was sutured with 3-0 Vicryl (polyglactin 910, Ethicon, Inc., Somerville, NJ, USA). Bilateral oophorectomy was carried out 1week after the first surgery in all groups for histological scoring (right ovaries) and biochemical evaluation (left ovaries). A 1-mL blood sample was obtained from the vena cava of each rat for the measurement of biochemical markers. After the completion of the overdose of anesthetic drugs, all rats were sacrificed.

### 2.3. Histopathological examinations

Ovarian tissues were fixed in 10% formalin for 48 h. After fixation, the tissues were prepared using standard procedures and embedded in paraffin. Serial 5-μm sections were cut from ovary blocks with a microtome and stained with hematoxylin–eosin. For each specimen, all sections were analyzed using a light microscope with 10× magnification (Olympus BX46; Olympus Corporation, Tokyo, Japan). Ovarian damage, including follicular cell degeneration, vascular congestion, hemorrhage, and inflammation (neutrophil infiltration), was scored histologically using a graduated scale (0 = none; 1 = mild; 2 = moderate; 3 = severe).Total scores were calculated according to these parameters. Evaluation was performed by a pathologist who was blind to the study groups. 

### 2.4. Biochemical analysis

Total antioxidant status (TAS) and total oxidant status (TOS) levels were measured using a colorimetric method applied by Erel [23]. The results are expressed in millimoles Trolox equivalent/L (mmol Trolox equivalent/g wet tissue) for TAS, and micromolar hydrogen peroxide (H2O2) equivalent per liter of TOS (μmol H2O2 equivalent/g wet tissue) [24].The ratio of TOS to TAS was accepted as oxidative stress index (OSI). For calculation, the miilimole TAS unit was first converted to a micromole unit. OSI value was calculated according to the following formula: OSI (arbitrary unit) = [TOS *μ *mol H2O2 equivalent*/*gr protein)*/*TAS *μ *mol Trolox equivalent*/*gr protein × 10]. The peroxynitrite assay and protein content were determined, as previously described [25,26]. The results were expressed as micromole/L for serum and micromole/g for wet tissue, respectively. Biochemical measurements were carried out using a spectrophotometer (Shimadzu U 1601, Shimadzu Corp., Kyoto, Japan).

### 2.5. Statistical analysis

The IBM Statistical Package for the Social Sciences (SPSS), version 24.0 (IBM Corp., Armonk, NY, USA) was used for statistical analysis. A two-tailed Pvalue of <.05 was considered significant. The mean, standard deviation, median (interquartile range), and sample size values for the variables are presented in the tables. The normality of the distribution of the variables was tested with the Shapiro–Wilk test. The homogeneity of the variances of the groups was tested with Levene’s test. Parametric variables were compared by one-way ANOVA, followed by Tukey’s HSD test. The multigroup comparison of nonparametric variables was conducted using the Kruskal–Wallis test with a two-tailed P-value significance threshold of 0.05. Pairwise comparisons were conducted using the Mann–Whitney U-test with Bonferroni correction. The correction was carried out by dividing the significance value of 0.05 by the total comparison number of the group, leading to a new adjusted significance level of 0.05/15 = 0.003.

## 3. Results

**Figure 1:**
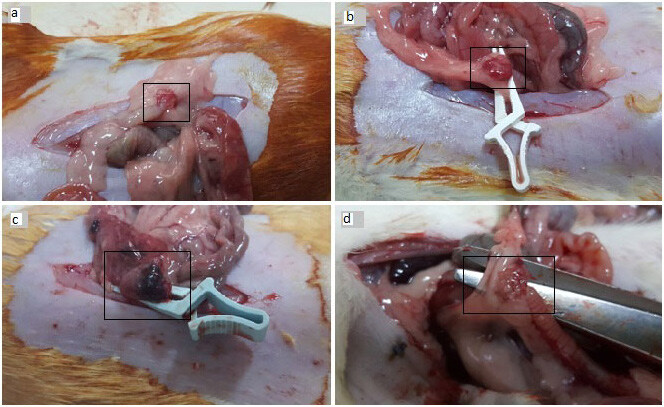
Surgical steps of addressing rat ovarian torsion model: a) rat ovary just before torsion, b) rat ovary just after torsion using bulldog vascular clamps, c) rat ovary with black-bluish appearance just before detorsion proceeding 3 h of ischemia, d) rat ovary 1 week after the detorsion.

With 8 rats in each of the 6 groups, a total of 48 rats were involved in the study. One rat in Group 4 died during the study. The macroscopic appearance of surgical procedures of rat ovarian torsion/detorsion model was as shown in Figure 1: Ovaries just before (1a) and after torsion (1b), and just before (1c) and 1 week after (1d) detorsion. 

The comparison of TAS, TOS, OSI, and peroxynitrite tissue levels between the sham (Group 1), surgical control (Group 2), ethanol control (Group 3), and melatonin treatment (Groups 4, 5, and 6) groups are shown in Table 1. Although tissue TAS, TOS, and OSI levels were improved in treatment groups, they did not differ significantly between the treatment and control groups. However, tissue levels of TOS in Group 1 compared to Group 3 (P = 0.0030) and peroxynitrite in Group 6 compared to Group 2 were significantly lower (P = 0.0040).

**Table 1:** Comparison of tissue TAS, TOS, OSI, and peroxynitrite levels between control and melatonin treated groups.

	TAS	TOS	OSI	Peroxynitrite
Group 1 (n: 8)	8.04 ± 1.36	83.59 ± 14.61	1.08 ± 0.34	38.18 ± 8.07
Group 2 (n: 8)	8.43 ± 1.83	114.80 ± 27.07	1.41 ± 0.38	45.34 ± 5.70
Group 3 (n: 8)	9.30 ± 2.65	120.38 ± 25.75	1.40 ± 0.58	44.15 ± 3.37
Group 4 (n: 7)	10.34 ± 1.12	106.89 ± 21.48	1.05 ± 0.25	34.77 ± 8.59
Group 5 (n: 8)	10.46 ± 1.76	100.02 ± 21.13	0.97 ± 0.24	35.20 ± 8.44
Group 6 (n: 8)	10.63 ± 1.83	108.20 ± 15.22	1.05 ± 0.23	34.01 ± 8.63
P-value	0.024a	0.029b	0.056b	0.009a

Values are presented as mean ± SD, median (interquartile range), T: tissue, TAS: total antioxidant status, TOS: total oxidant status; OSI: oxidative stress index, n; number Units of ovarian tissue TAS: millimol Trolox Eq/gr, TOS: micromol H2O2 Eq/gr, OSI: arbitrary unit, and Peroxynitrite: micromol/gr. P-values were calculated with aone-way ANOVA or bKruskal–Wallis test. The significant pairwise group comparison results were as represented below: For TAS: no significant pairwise comparison (post hoc test: Tukey HSD). For TOS: 1 vs. 3: P = 0.0030 (Mann–Whitney U test with Bonferroni correction). For peroxynitrite: 2 vs. 6: P = 0.040 (post hoc test: Tukey HSD)

Table 2 shows the comparison of serum TAS, TOS, OSI, and peroxynitrite levels among the groups. Group 2 had significantly higher serum levels of TOS and peroxynitrite than Groups 1 and 6 (both P = 0.0023). Moreover, serum OSI levels were significantly increased in Group 2 when compared to Groups 1 and 6 (P = 0.0023 and P = 0.0016, respectively), and in Group 3 compared to Groups 1, 4, 5, and 6 (P = 0.0023, P = 0.0026, P = 0.0008 and P = 0.0011, respectively).

**Table 2:** Comparison of serum TAS, TOS, OSI, and peroxynitrite levels between control and melatonin treated groups.

	TAS	TOS	OSI	Peroxynitrite
Group 1 (n: 8)	1.12 ± 0.13	10.37 ± 6.80	0.96 ± 0.67	0.85 ± 0.20
Group 2 (n: 8)	1.04 ± 0.07	26.64 ± 10.12	2.56 ± 1.01	1.28 ± 0.24
Group 3 (n: 8)	1.21 ± 0.42	9.12 ± 8.02	2.71 ± 0.56	1.16 ± 0.28
Group 4 (n: 7)	1.03 ± 0.07	24.31 ± 7.10	1.07 ± 0.52	0.75 ± 0.39
Group 5 (n: 8)	1.18 ± 0.07	14.68 ± 7.82	0.94 ± 0.36	0.91 ± 0.38
Group 6 (n: 8)	1.24 ± 0.20	13.99 ± 9.41	0.72 ± 0.49	0.60 ± 0.34
P-value	0.009	0.002	<0.001	0.002

Values are presented as mean ± SD, median (interquartile range), T: tissue, TAS: total antioxidant status, TOS: total oxidant status; OSI: oxidative stress index, n; number Units of serum TAS: millimol Trolox Eq/L, TOS: micromol H2O2 Eq/L, OSI: arbitrary unit and Peroxynitrite: micromol/L.P-values for multigroup comparisons were calculated with Kruskal–Wallis. The significant pairwise group comparison results were as represented below: (post hoc test: Bonferroni correction) For TAS: 4 vs. 5: P = 0.0026. For TOS: 1 vs. 2: P = 0.0023. For OSI: 1 vs. 2: P = 0.0023, 1 vs. 3: P = 0.0023, 2 vs. 6: P = 0.0016, 3 vs. 4: P = 0.0026, 3 vs. 5: P = 0.0008, 3 vs. 6: P = 0.0011. For peroxynitrite: 2 vs. 6: P = 0.0023.

Table 3 shows the comparison of histopathological scores for ovarian damage between the groups. All scores were significantly lower in Group 1 than in almost all other groups. Moreover, there were significant increase in histopathological scores of control groups regarding hemorrhage (Group 2 vs. Group 5: P = 0.0008; Group 3 vs. Groups 4, 5, and 6: P = 0.0014, P = 0.0002, P = 0.0008, respectively), congestion (Group 3 vs. Groups 5 and 6: P = 0.0008 and P = 0.0028, respectively) and follicular degeneration (Group 2 vs. Group 5: P = 0.0013, and Group 3 vs. Group 5: P = 0.0032) with respect to treatment groups.

**Table 3:** Comparison of histopathological scores of ovarian damage between control and melatonin treated groups.

	Hemorrhage	Congestion	Folliculardegeneration	Inflammation
Group 1 (n: 8)	0.00 ± 0.00	1.00 ± 0.00	0.13 ± 0.35	0.13 ± 0.35
Group 2 (n: 8)	1.88 ± 0.35	1.88 ± 0.64	2.38 ± 0.74	2.75 ± 0.46
Group 3 (n: 8)	2.13 ± 0.35	2.13 ± 0.35	2.00 ± 0.76	3.00 ± 0.00
Group 4 (n: 7)	1.14 ± 0.38	1.29 ± 0.76	1.00 ± 0.58	2.00 ± 0.82
Group 5 (n: 8)	0.88 ± 0.35	1.13 ± 0.35	0.75 ± 0.46	1.88 ± 0.83
Group 6 (n: 8)	1.13 ± 0.35	1.25 ± 0.46	1.13 ± 0.64	1.88 ± 1.13
P-value	<0.001	<0.001	<0.001	<0.001

Values are presented as mean ± SD, median (interquartile range),n: number. P-values were calculated with Kruskal–Wallis. The significant results by pairwise group comparison using Mann–Whitney U test with Bonferroni correction were as represented below: For hemorrhage: 1 vs. 2: P = 0.0001, 1 vs. 3: P = 0.0001, 1 vs. 4: P = 0.0002, 1 vs. 5: P = 0.0006, 1 vs. 6: P = 0.0001, 2 vs. 5: P = 0.0008, 3 vs. 4: P = 0.0014, 3 vs. 5: P = 0.0002, 3 vs. 6: P = 0.0008. For congestion: 1 vs. 2: P = 0.0031, 1 vs. 3: P = 0.0001, 3 vs. 5: P = 0.0008, 3 vs. 6: P = 0.0028. For full degeneration: 1 vs. 2: P = 0.0005, 1 vs. 3: P = 0.0006, 2 vs. 5: P = 0.0013, 3 vs. 5: P = 0.0032. For inflammation: 1 vs. 2: P = 0.0003, 1 vs. 3: P = 0.0001, 1 vs. 4: P = 0.0009, 1 vs. 5: P = 0.0007, 1 vs. 6: P = 0.0027.

Table 4 shows the comparison of rat ovarian follicle counts between control and melatonin-treated groups. Primary, secondary, and tertiary follicle counts tend to be higher in the sham (Group 1) and melatonin-treated groups (Groups 4, 5, and 6) when compared to the surgical control rats (Group 2), but the difference was not statistically significant. However, primordial follicle count was statistically significantly higher in Group 6 in comparison with Groups 2 (P = 0.0002), 3 (P = 0.0005), 4 (P = 0.0012), and 5 (P = 0.0027). 

**Table 4:** Comparison of ovarian follicle count between control and melatonin treated groups.

	Primordial	Primary	Secondary	Tertiary
	follicle count (n)	follicle count (n)	follicle count (n)	follicle count (n)
Group 1 (n: 8)	8.3 ± 3.4	5.9 ± 3.8	4.5 ± 1.6	8.4 ± 2.7
Group 2 (n: 8)	4.8 ± 1.5	3.9 ± 1.6	3.4 ± 1.7	3.6 ± 2.5
Group 3 (n: 8)	5.1 ± 1.7	5.9 ± 2.2	4.8 ± 1.2	7.0 ± 1.4
Group 4 (n: 7)	5.3 ± 1.4	4.0 ± 1.4	4.4 ± 1.6	7.9 ± 3.5
Group 5 (n: 8)	5.8 ± 1.3	3.9 ± 1.0	4.9 ± 2.0	6.6 ± 3.3
Group 6 (n: 8)	10.3 ± 3.0	5.3 ± 2.1	5.3 ± 2.8	8.1 ± 2.4
p-value	<0.001a	0.263b	0.584b	0.023b

Values are presented as mean ± SD, median (interquartile range), n: number.P-values were calculated with aone-way ANOVA or bKruskal–Wallis test. The significant pairwise group comparison results were as represented below: For primordial follicle count: 2 vs. 6: P = 0.0002, 3 vs. 6: P = 0.0005, 4 vs. 6: P = 0.0012, 5 vs. 6: P = 0.0027 (post hoc test: Tukey HSD) For tertiary follicle count: 1 vs. 2: P = 0.003 (Mann–Whitney U test with Bonferroni correction)

**Figure 2:**
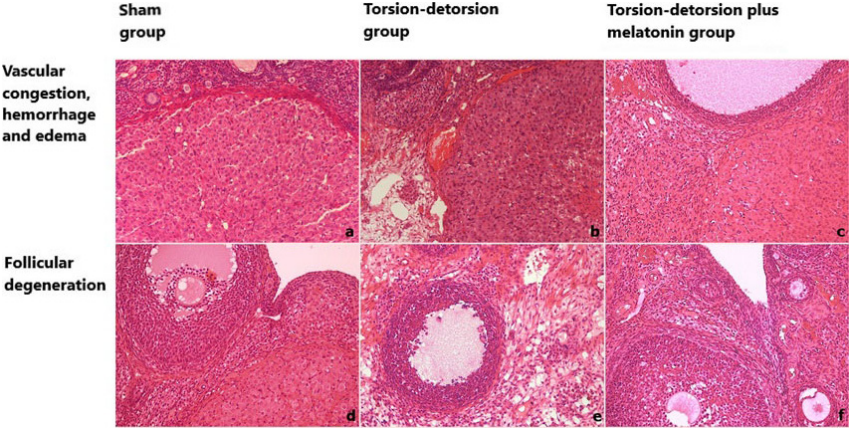
Histological sections of rat ovaries stained with hematoxylin and eosin (H&E): a) normal ovarian morphology with normal ovarian cortical and follicular architecture in the sham group (Group 1), H&E×200, b) vascular congestion, hemorrhage and edema increased in the torsion/detorsion group (Group 2), H&E×200, c) mild vascular congestion hemorrhage and edema in the torsion/detorsion + melatonin group (Group 5), H&E×200, d) ovarian follicle without follicular degeneration in sham group (Group 1), H&E×200, e) ovarian follicle with prominent follicular degeneration in torsion/detorsion group (Group 2), H&E×200, f) ovarian follicle with mild follicular degeneration in torsion/detorsion + melatonin group (Group 6), H&E×200.

Figure 2 shows histological sections of rat ovaries. Vascular congestion, hemorrhage, and edema were normal in the sham group (2a), much increased in the surgical control group (2b), and mildly increased inthe melatonin group (2c). Similarly, follicular degeneration was absent in the sham group (2d), much increased in the surgical control group (2e), and mildly increased in the melatonin group (2f).

## 4. Discussion

The present study focused on the effects of melatonin on ovarian ischemia reperfusion injury in a surgically-induced torsion/detorsion model of rats by modulating oxidant and antioxidant status. At the end of the study, it was found that melatonin signiﬁcantly decreased the ischemia reperfusion injury of the ovary both histopathologically and biochemically. There were signiﬁcant reductions in both tissue and serum levels of peroxynitrite, while serum OSI level was signiﬁcantly decreased in the melatonin group when compared to control rats. Moreover, primordial follicle count was statistically significantly higher in the melatonin-treated group than in the surgical control group. Furthermore, histopathological analysis showed that the ovaries of the rats treated with melatonin had signiﬁcantly lower hemorrhage, follicular degeneration, and total tissue damage scores than those of rats in the control groups.

Ovarian torsion is a painful gynecologic emergency with a prevalence of about 3%; arterial or venous blockage may come out in the adnexa as a result of this rotation. Immediate detorsion is the main treatment to solve ischemia and establish reperfusion. Ovarian ischemia and reperfusion during the torsion/detorsion process may cause oxidative stress through overproduction of ROS in a pathologic amount. Reperfusion injury after detorsion has been reported to be related with excessive release of oxygen species [27,28]. Thus, prevention of ovarian damage caused by ischemia reperfusion injury is important in the area of gynecology.

Numerous therapeutic agents showing antioxidant actions have been studied for protective effects against ovarian ischemia reperfusion injury rat models [29–32]. Melatonin, an endogenous indolamine related to circadian rhythms, has proven itself as an essential agent against toxic free radicals and provides a strong defense against ischemia reperfusion injury [33,34]. It was found in our previous study that melatonin caused regression of endometriotic implants in rats by improving antioxidation actions on superoxide dismutase and malondialdehyde [17].

TAS and TOS reﬂect the total effects of all antioxidants and oxidants in the plasma, body ﬂuids, or tissues, respectively [23]. OSI is calculated with the formula of TOS/TAS and is regarded as a more appropriate index of oxidative stress on the plasma or tissue. Several antioxidant free radical scavengers have been shown to prevent ovarian ischemia reperfusion injury in rats by improving TAS, TOS, and OSI [35,36]. Peroxynitrite (ONOO–) is the major component of nitroxidative stress. When reactive nitrogen species are overproduced, nitric oxide (NO•) reacts quickly with the superoxide radical (O2•–) to form peroxynitrite (ONOO–), which is itself cytotoxic, and then easily decomposes into toxic hydroxyl radical (OH •) and nitrogen dioxide. As peroxynitrite exerts its cytotoxic action thru mitophagy, inhibiting peroxynitrite-mediated mitophagy activation improves ischemia reperfusion injury [37,38]. Moreover, melatonin as an antioxidant improves TOS, TAS, OSI, and peroxynitrite, as demonstrated in previous studies [39–41].

To the best of our knowledge, protection of the rat ovary from ischemia reperfusion injury using melatonin has been investigated in only 3studies up to date. In thefirst study, histopathological scores and malondialdehyde were significantly improved by melatonin in a rat torsion/detorsion model [18]. In the second study, melatonin (10 mg/kg i.p. for 3 h) was found to protect the ovaries against oxidative damage associated with reperfusion following an ischemic insult in rats, based on the histopathological and biochemical ﬁndings including malondialdehyde, glutathione, and xanthine oxidase [19]. Similarly, malondialdehyde, myeloperoxidase, glutathione, glutathione peroxidase, glutathione reductase, glutathione s-transferase, and superoxide dismutase were found to be significantly improved by 2.2 mg/kg i.p. melatonin in the third study [20]. Moreover, it has been reported that melatonin decreased cat ovarian [21] and rat uterine [22] tissue injury after detorsion.

In our current study, rats were divided into 6 groups: the sham group; the torsion/detorsion group; 3 melatonin (10 mg/kg i.p.) groups (only before torsion as a single dose in the prophylaxis group; only after detorsion daily for 1 week in the maintenance group; and both before torsion and after detorsion daily for 1 week in the prophylaxis plus maintenance group, respectively). 

The rationale behind scheming our melatonin groups as the prophylaxis, maintenance, and prophylaxis plus maintenance groups in our study was to mimic real clinical scenarios in which a patient attends with the suspicion of adnexal torsion, (i) the physician can give the therapeutic agent before detorsion for prophylactic treatment, (ii) the physician can give the therapeutic agent only after detorsion as a maintenance treatment, or (iii) the physician can give the therapeutic agent not only before detorsion for prophylactic treatment, but also daily after detorsion for a while for maintenance treatment (prophylaxis plus maintenance treatment). However, there was no significant difference between these treatment groups.

The results of this study have shown that melatonin reduced ischemia reperfusion damage in the rat ovary by histopathological and biochemical parameters as shown for the third time in the literature. However, our study is the first in the literature to show that melatonin prevented ischemia reperfusion in a rat ovarian torsion/detorsion model by improving oxidative stress in both tissue and serum levels of peroxynitrite and serum levels of OSI. Moreover, the melatonin group had a significantly higher primordial follicle count than the surgical control group.

This study has several limitations. An experimental rat model of ovarian torsion/detorsion cannot really exemplify the classical pathophysiology in clinical practice in humans. In addition, there is no consensus on the optimal dose and optimal duration of treatment with melatonin. Moreover, a 7-day follow-up period corresponds to a length of approximately 2 menstrual cycles, as the estrous cycle of the rats takes about 4–5 days. However, it would be improved if the rats were followed up with for a longer period of time. Furthermore, it would be better to measure primordial follicle count to see the effects of ischemia reperfusion injury. Rotation of the pedicle may not be needed to induce ischemia reperfusion injury after vascular clip usage, as it might have caused vascular injury in this experimental model. Additionally, intraperitoneal alcohol is known as a sclerosing agent, but it was demonstrated that even intraperitoneal 99.9% ethanol had no more severe side effects [42]. Moreover, the ethanol used in this study was only 5% in concentration. Thus, further prospective animal and even human studies are needed to determine the value of melatonin for protecting ovaries from ischemia reperfusion injury after detorsion.

All in all, our results show that melatonin exerts protective effects against ischemia reperfusion injury in a rat torsion/detorsion model by improving histopathological and biochemical findings, including primordial follicle count, tissue and serum levels of peroxynitrite, and serum levels of oxidative stress index.

**Conflict of interest**

The authors report no conflict of interest with any funding agency or commercial sectors.
